# The Mental Health Implications of People‐Pleasing: Psychometric Properties and Latent Profiles of the Chinese People‐Pleasing Questionnaire

**DOI:** 10.1002/pchj.70016

**Published:** 2025-05-01

**Authors:** Xiaoxue Kuang, Hui Li, Weiliang Luo, Jinxin Zhu, Fen Ren

**Affiliations:** ^1^ School of Education Dongguan University of Technology Dongguan China; ^2^ School of Education Guangzhou University Guangzhou China; ^3^ Nancheng Converged Media Center Guangzhou China; ^4^ Department of Curriculum and Instruction Faculty of Education and Human Development, The Education University of Hong Kong Hong Kong SAR China; ^5^ School of Education and Psychology University of Jinan Jinan China

**Keywords:** construct validity, latent profile analysis, measurement invariance, mental health, people‐pleasing

## Abstract

This study explores the mental health implications of people‐pleasing behaviors by validating the 24‐item Chinese People‐Pleasing (CPP) questionnaire with a sample of 2203 Chinese university students. A three‐factor structure—comprising thought, behavior, and feeling dimensions—was confirmed after the removal of 11 items. The revised 13‐item CPP demonstrated good model fit, strong internal consistency, and satisfactory construct validity. Measurement invariance analysis revealed consistent results across gender, academic disciplines, and birthplace, as well as strong longitudinal invariance. These findings provide support for the validity and reliability of the 13‐item CPP as an assessment tool within the Chinese cultural context. Furthermore, latent profile analysis revealed four distinct profiles of people‐pleasing tendencies, which varied from mild to severe. Notably, higher people‐pleasing tendencies were significantly associated with lower levels of mental well‐being, highlighting its potential impact on students' psychological health. These insights emphasize the potential clinical utility of CPP in addressing mental health concerns associated with people‐pleasing behaviors, particularly in the Chinese cultural context.

## 1 Introduction

People‐pleasing involves prioritizing the needs and desires of others over one's own needs and desires (Ooms 2023). This behavior appears among individuals who exert significant effort to satisfy others, often at the expense of their own well‐being (Ooms 2023). Chronic people‐pleasing has been linked to heightened neuroticism, anxiety, and emotional exhaustion, potentially leading to mental health disorders (Joiner 2005). However, in the Chinese cultural context where collectivist values emphasize harmony and interpersonal relationships, the desire to please others can easily become a important way for people to socialize and even be seen as a vital skill for social survival. This overwhelming urge to satisfy others may also impose considerable psychological pressure. Therefore, recognizing one's tendency for people‐pleasing (TPP) can help individuals develop a deeper understanding of themselves and make more conscious choices, rather than automatically seeking to please others (Braiker 2001), and a valid screening instrument is needed for people‐pleasing in the Chinese context (Ehman 2021; Ou et al. 2021). This study aims to address this issue by investigating the psychometric properties of a Chinese version of the people‐pleasing questionnaire (CPP) with a large sample of university students in China.

### 1.1 People‐Pleasing and Its Negative Effects

To people‐pleasers, meeting others' needs is often seen as the magic formula for gaining love and self‐worth or avoiding abandonment and rejection (Braiker 2001). Many people‐pleasers are driven by the belief that being agreeable can shield them from uncomfortable conflicts, particularly within their social circles or family (Braiker 2001). For them, saying “no” to others can be a significant challenge, as they view compliance with requests as a way to secure a sense of safety against potential rejection or isolation (Ooms 2023). Some people‐pleasers may also experience apprehension about harming others' feelings or the possibility of disappointing them when declining requests. Consequently, people‐pleasers often struggle to articulate their own needs, choosing instead to remain silent in pursuit of external approval (Ehman 2021; Svoboda 2008).

However, people‐pleasing behaviors may result in unfavorable outcomes, especially when the expectations for acceptance from others are not fulfilled. Specifically, individuals who engage in people‐pleasing behavior may exhibit a high level of agreeableness (i.e., a tendency to be friendly, cooperative, and considerate when interacting with others) and neuroticism (i.e., a tendency to experience negative emotions such as anxiety and self‐doubt with greater intensity and frequency) (Bagby et al. 2001). These individuals may also be more susceptible to mental health disorders, including anxiety and depression, due to the emotional strain caused by their excessive desire to please others (Trull and Widiger 2013). People‐pleasing behaviors are also associated with emotional exhaustion, as individuals take on additional responsibilities and tasks to gain others' approval (Ehman 2021), which can have a detrimental effect on their overall well‐being (Robins et al. 1994).

Further, the relationship between TPP and the big five personality traits is noteworthy. People‐pleasers typically exhibit high levels of agreeableness, but this can be coupled with high neuroticism, leading to difficulties in regulating emotions and experiencing greater psychological distress. People‐pleasing can also be linked to feelings of solitude and loneliness, as individuals may suppress their authentic selves in favor of accommodating others, leading to shallow or unsatisfying social connections (Cacioppo and Patrick 2008). Additionally, people‐pleasers often have lower core self‐evaluations, which include self‐esteem, self‐efficacy, and emotional stability, as their sense of worth is largely dependent on external validation (Judge et al. 2003). This over‐reliance on others' approval can undermine mental well‐being, contributing to feelings of emptiness and dissatisfaction (Neff 2003). In sum, while people‐pleasing behaviors may be rooted in an attempt to secure social harmony and acceptance, they can have significant negative consequences for mental health, especially when they interfere with an individual's ability to establish authentic relationships, maintain emotional balance, and foster a positive sense of self.

### 1.2 People‐Pleasing in the Chinese Context

In Chinese Mainland, shaped by a collectivist culture, the concept of interpersonal harmony is deeply ingrained, prioritizing collective well‐being and social cohesion over individual desires. Within this context, people‐pleasing is often viewed as a pro‐social trait, celebrated in mainstream society as a hallmark of being a “good person”. It is also considered a key social skill, embodying the essence of “worldly wisdom” and effective relationship management (Wang 2007). Building strong interpersonal relationships and social networks is highly valued in Chinese society, both personally and professionally. Through people‐pleasing behaviors, individuals often enhance their social connections, gain crucial support, advance their careers, and improve their overall quality of life. People‐pleasing tendencies are particularly prominent among Chinese university students. For many students, university marks the first time they leave the familial environment to navigate complex social dynamics independently. These students may struggle to decline requests due to fears of social isolation, a desire for approval, or a need for harmonious interactions. Consequently, they often prioritize others' needs at the expense of their own, leading to excessive people‐pleasing behaviors. This over‐accommodation can result in feelings of inferiority, avoidance of interpersonal relationships, and negative emotions, ultimately hindering their academic performance and personal growth (Ma 2020). It is thus important to have a tool to measure the inclination toward excessive people‐pleasing. This tool can assist individuals in better understanding their position within their social circles and help them strike a healthier balance between their own needs and the expectations of others. It also enables more effective management of interpersonal relationships. Employing such a tool to assess TPP among university students can also build a scientific foundation for relevant mental health education.

## 2 Measurement of People‐Pleasing

### 2.1 Previous Measurements for People‐Pleasing

Earlier studies on people‐pleasing often employed qualitative labels to define people‐pleasers and used the term as a potential variable to explain other concepts such as therapeutic processes, therapeutic action, anxiety, depression mechanisms, and other disorder behaviors (Ehrenberg 2012). Kushner and Choi (2010), for example, investigated the prevalence of unhealthy eating, exercise habits, and coping pattern traits among overweight and obese adults, and being a “people‐pleaser” was identified as one of the coping patterns. This pattern was found to be systematically influenced by Body Mass Index (BMI, kg/m^2^). People‐pleasers tended to overeat in social environments and were more likely to have disordered eating habits or eating disorders (Exline et al. 2012). However, it is noteworthy that in prior studies, the term “people‐pleaser” was directly used to describe a personal trait, but a clear definition and psychometric indicators for assessment were lacking. A recent YouGov survey of 1000 adult American citizens showed that women (56%) were more likely to self‐identify as being a people‐pleaser than men (42%), and Democrats (57.8%) were more likely to identify as such compared to members of other political parties (48.6%) (YouGov 2022). It is important to note that the survey relied on a single self‐reported item to assess people‐pleasing behavior.

### 2.2 People‐Pleasing as a Personality Type

While some research has used the previous measurements, other researchers have treated people‐pleasing as a personality type within a classification system. For example, in the classification of Kefir (1981), pleasers considered to be people who wish to avoid situations in which they experience rejection. Pew (1976) identified pleasers as individuals who strive to connect with others by ensuring the happiness of those important to them. Later, Langenfeld and Main (1983) developed the Langenfeld Inventory of Personality Priorities (LIPP), a tool that measures personality priorities, including pleasing, through 15 specific items. According to LIPP, people‐pleasers are characterized by their desire to make others happy and gain acceptance. They seek this acceptance primarily by fulfilling others' needs, desires, and expectations. The exploratory factor loading of pleasing ranged between 0.31 and 0.63. No precise information about reliability was provided in Langenfeld's study. Numerous studies have used LIPP to examine the differences between different personality types in terms of self‐esteem, social interest, locus of control, dysfunctional attitudes, wellness orientation, depression, self‐efficacy, and positive affect (e.g., Ashby and Kottman 2000). However, these studies often treated “being a pleaser” as a single‐dimensional trait within a test, overlooking the complexity and profound impact that this behavior can have on individuals.

### 2.3 Psychometric Properties of Existing Established Instruments

Braiker (2001) developed a 24‐item quiz to assess an individual's TPP. The participants were instructed to indicate whether each item was “true” (coded as 1) or “false” (coded as 0). The cumulative score from these responses is an indicator of an individual's people‐pleasing inclination. This assessment categorized individuals into three types of people‐pleasers: cognitive, behavioral, and emotionally avoidant. People‐Pleasing has three core components: distorted thought patterns (mindsets), repetitive compulsive behaviors (habits), and heightened fear‐based emotions (feelings). If a participant's total score falls within the range of 16–24, it indicates a severe manifestation of people‐pleasing syndrome. Scores between 10 and 15 suggest a moderately severe presentation, while scores from 5 to 9 signify a moderate tendency to please others. Scores below 4 indicate a mild form of TPP. However, it is important to note that there is currently no empirical evidence to confirm the reliability or validity of Braiker's questionnaire. Additionally, the cutoff criteria for identifying individuals with people‐pleasing syndrome lack sufficient empirical support.

Ehman (2021) introduced an approval rating test as a tool for assessing TPP behavior. The test comprises 20 items, including statements like, “If I sense that someone does not like me, it bothers me.” Participants rate their responses on a Likert scale ranging from 1 (*always*) to 5 (*never*). The total score, calculated by summing the ratings, reflects the extent of an individual's TPP Scores of 81–100 indicate no self‐sabotaging behavior due to excessive people‐pleasing. Scores between 61 and 80 represent an average level of TPP, while scores of 41–60 suggest minor issues. Participants scoring 31–40 exhibit symptoms of people‐pleasing syndrome, and scores of 21–30 indicate a serious manifestation. A score of 20 signifies a severe case requiring intervention. However, as with Braiker's questionnaire, this scale lacks empirical evidence to support its cutoff criteria for diagnosing people‐pleasing syndrome. Additionally, the reliability and validity of Ehman's quiz remain unverified, limiting the generalizability and robustness of conclusions drawn from its use.

Ou et al. (2021) developed a 28‐item questionnaire designed to assess people‐pleasing behavior within the Chinese context. The questionnaire includes six dimensions: compromise and sacrifice, inferiority and inhibition, kindness and deference, empathy, compliment and pandering, and interpersonal sensitivity. However, the six‐factor structure, evaluated through confirmatory factor analysis (CFA), demonstrated poor model fit. Key fit indices, such as the comparative fit index (CFI = 0.79) and Tucker–Lewis index (TLI = 0.76), fell significantly below the acceptable threshold of 0.90. Consequently, the validity of the proposed factor structure remains questionable.

The research conducted by these three scholars/teams has yielded three distinct measurement tools for assessing TPP. Braiker's tool has been adopted in this study because it was the first one to highlight this issue and the tool incorporates clear theory construction based on many years of clinical experience. Unlike other existing measures, Braiker's model delineates three core aspects of people‐pleasing behavior—thoughts, behaviors, and feelings—providing a comprehensive understanding of the construct. Furthermore, it is deeply rooted in clinical observations and psychological theory, making it a robust instrument for exploring the psychological consequences of people‐pleasing. In addition, Jiang (2015) translated Braiker's scale into Chinese version, which can be directly used for this study (Blake 2015); however, the psychometric properties of this localized instrument, including its reliability and validity, remain unverified. Moreover, neither the original questionnaire nor Jiang's translation underwent empirical validation or cultural adaptation for the Chinese context. Therefore, this study aimed to refine and validate Jiang's version to enhance its applicability in mental health research within collectivist societies.

The primary aim of this study was thus to assess the psychometric properties of Jiang's translated Chinese version of Braiker's people‐pleasing questionnaire (CPP) with a sample of university students in Chinese Mainland. This adaptation and evaluation provide valuable insights into the relevance of Braiker's tool within this unique cultural context, as well as contributing to a deeper understanding of TPP among Chinese university students. This study therefore sought to establish cutoff criteria for individuals exhibiting TPP using contemporary statistical methods such as latent profile analysis (LPA). Three key questions were addressed in this study:

1. Is CPP suitable for application in a Chinese context?
2. What are the reliability and validity metrics of CPP in the Chinese context?
3. Can LPA be employed to establish cutoff criteria for identifying individuals with TPP?

## 3 Method

### 3.1 Procedure

The study was conducted in three phases: pre‐testing, formal testing, and follow‐up. In the pre‐testing phase, 602 first‐year university students completed the original 24‐item CPP. Based on exploratory factor analysis (EFA), six items were removed to improve construct validity. In the formal testing phase, 2498 first‐year students completed the revised version, and CFA was conducted to assess the internal structure and reliability of the scale. One month later, 2340 students participated in the follow‐up study, completing the revised TPP questionnaire along with additional psychological measures to examine criterion‐related validity. The collected data were analyzed using SPSS27.0 and Mplus8.0 software to ensure statistical robustness.

### 3.2 Sample

The data used in this study were obtained with two cohorts from a university located in Chinese Mainland. Data collection commenced in September 2022 and concluded in July 2023. All surveys received individual approval from the Research Ethics Board of Dongguan University of Technology. All participants provided informed consent prior to participation in the study. The testing process was conducted in three stages: the first stage involved the pre‐testing phase, followed by formal testing in Cohort 1, with a subsequent follow‐up in Cohort 2 1 month later.

In the pretesting phase, we enrolled 602 first‐year students (319 male, 283 female) and administered a 24‐item CPP.

In Cohort 1, we enrolled 2498 first‐year students (1362 male, 1136 female) and administered a modified version of the CPP questionnaire, along with background information.

One month later, in Cohort 2, 2340 students (1262 male, 1078 female) were asked to complete the TPP questionnaire again. In addition, they were assessed on the Big Five personality traits (Zhang et al. 2019), self‐evaluation (Judge et al. 2003), and a solitude scale (Chen 2007), which were used as criterion variables. The Warwick‐Edinburgh Mental Well‐being Scale (Dolan and White 2010) was also administered, serving as a predictive variable. Both cohorts completed the tests in the classroom, and all data were collected through a university‐developed online application. The average response time was 235 s. After excluding participants who answered less than 25% of the questions and spent less than 60 s, the final sample for the study comprised 2203 students in both cohorts, with 44.45% (*n* = 978) being female and 55.55% male. All students were in their first year. Out of the total sample, 1138 participants were from urban areas (51.7%), while 1065 were from rural areas (48.3%). Additionally, 1174 participants majored in science (53.3%) and 1029 in arts (46.7%).

### 3.3 Measures

#### 3.3.1 People‐Pleasing

The original CPP consists of 24 items, structured across three dimensions: thoughts related to people‐pleasing, behaviors associated with people‐pleasing, and feelings connected to people‐pleasing. Sample items include statements like: “I find myself compelled to do things for others to make them happy.” In this study, participants were instructed to rate their responses on a Likert scale that ranged from 1 (*completely untrue*) to 6 (*completely true*).

The Cronbach's alpha coefficient for the whole 24‐item version scale was 0.88.

The Cronbach's alpha coefficients for TPP thoughts, behaviors, and feelings were 0.71, 0.80, and 0.71, respectively. Model fit for the three factor structure of the previous 24‐item was unacceptable (CFI = 0.817, TLI = 0.797, RMSEA = 0.07, SRMR = 0.07). Through EFA on the pre‐testing sample (*n* = 602) using SPSS 27.0, two items were removed due to factor loadings below 0.4 (Elliott et al. 2021; Liu et al. 2024). These items were: “I rarely delegate tasks to others” and “I go to great lengths to avoid conflict or confrontation with my family, friends, or coworkers.” Another four items were removed due to cross‐loading issues: “I believe that nice people get the approval, affection, and friendship of others,” “I have to give of myself all the time in order to be worthy of love,” “It is extremely important to me to be liked by nearly everyone in my life,” and “I would think that I am a bad person if I didn't give of myself all the time.” The content of these items was also reviewed by a psychological scholar, an educational scholar, and five college students, all of whom recommended their removal. In the Chinese context, most students prioritize their academic commitments, making it unrealistic for them to dedicate all their time solely to others. For example, the item “I rarely delegate tasks to others” was deemed not directly related to TPP, as it could instead reflect a lack of leadership responsibility. In a culture that emphasizes collectivism and social harmony, behaviors such as avoiding conflict and seeking approval are common, but they do not necessarily reflect TPP. Consequently, the six items were removed. As a result, the original 24‐item CPP scale was revised to an 18‐item version CPP.

#### 3.3.2 Big Five Personality

The short version of the Big Five Personality Inventory was employed, comprising five dimensions: openness, agreeableness, conscientiousness, extraversion, and neuroticism. Each dimension was measured by three items (Zhang et al. 2019). Sample items include statements like “I like to take adventures.” Participants rated their responses on a Likert scale spanning from 1 (*strongly disagree*) to 5 (*strongly agree*). The reliability of the openness, agreeableness, conscientiousness, extraversion, and neuroticism dimensions in our study was 0.822, 0.844, 0.806, 0.905, and 0.883, respectively.

#### 3.3.3 Core Self‐Evaluation

The core self‐evaluation scale employed in this study consisted of 12 items (Judge et al. 2003). Sample items include statements like “I am confident that I achieve the success I deserve in life.” Participants provided responses on a Likert scale, ranging from 1 (*strongly disagree*) to 5 (*strongly agree*). The Cronbach's alpha coefficient for the scale in our study was 0.899.

#### 3.3.4 Solitude

Solitude was measured using a 16‐time scale developed by Chen (2007) in Chinese with four dimensions: positive solitude (e.g., “I can engage in activities that genuinely interest me when I'm alone”), eccentricity (e.g., “I prefer being alone and have little interest in others”), social avoidance (e.g., “I feel anxious when conversing with unfamiliar people”), and loneliness (e.g., “I experience loneliness when I have no company”). Participants were asked to express their level of agreement with the 16 statements using a 5‐point scale that ranged from 1 (*strongly disagree*) to 5 (*strongly agree*). A high score indicated a high level of positive solitude, eccentricity, social avoidance, or loneliness. In terms of reliability, the Cronbach's alpha coefficients of the four dimensions were 0.891 for positive solitude, 0.842 for eccentricity, 0.876 for social avoidance, and 0.848 for loneliness. The social avoidance and loneliness was used as criterion variables in this study.

#### 3.3.5 Mental Well‐Being

The Warwick‐Edinburgh Mental Well‐being Scale employed in this study comprises 14 items (Dolan and White 2010). Example items include statements such as “I've been feeling optimistic about the future.” Participants provided their responses rated using a Likert scale, ranging from 1 (*never*) to 5 (*always*). The Cronbach's alpha coefficient in this study was 0.950.

### 3.4 Statistical Analysis

The entire sample of 2203 participants in Cohort 1 was randomly divided into two halves. The first half of the sample (*n* = 1102) was used for EFA and item analysis, while the second half (*n* = 1101) was employed to conduct CFA to assess the internal structure and validity of the scale using Mplus 8.0.

To determine the model fit, several indices were employed, including Chi‐square statistics, the CFI, TLI (good if CFI or TLI > 0.90; better if CFI or TLI > 0.95) (Byrne 2012; Hu and Bentler 1999), root mean square error of approximation (RMSEA; good if RMSEA < 0.05; acceptable if values ranging from 0.05 to 0.08; Maccallum et al. 1996), and the standardized root mean square residual (SRMR; acceptable if ≤ 0.08). Factor loadings were interpreted as follows: poor (< 0.32), fair (> 0.40), good (> 0.50), very good (> 0.60), and excellent (> 0.70) (Tabachnick and Fidell 2013).

Internal consistency was assessed using Cronbach's alpha as calculated with SPSS27.0, considering values above 0.80 as indicative of good consistency and values below 0.50 as unacceptable (Nunnally 1978). Additionally, the composite reliability was evaluated using McDonald's (1970) *ω*, with values exceeding 0.70 reflecting adequate composite reliability (Dunn et al. 2014), a more robust measure than Cronbach's α (McNeish 2017).

To evaluate test–retest reliability, intra‐class correlation coefficients were calculated, with values below 0.50 indicating poor reliability, 0.50–0.75 signifying moderate reliability, 0.75–0.90 representing good reliability, and values exceeding 0.90 indicating excellent reliability (Koo and Li 2016).

The Average Variance Extracted (AVE) and its square root (SQRAVE) were used to assess convergent and discriminant validity. AVE represents the average amount of variance a latent variable captures from its observed indicators, which is calculated by averaging the squared factor loadings of the indicators for each latent variable. AVE values greater than 0.5 indicate good convergent validity. SQRAVE is the square root of the AVE value, which ensures that the latent variables are distinct from each other. If the SQRAVE value is greater than the correlations between the latent variable and any other latent variable, it indicates good discriminant validity (Hair et al. 2017).

Convergent validity was evaluated by correlating its average scores with the average CPP scores using Pearson correlation. The CPPcriterion‐related validity was evaluated by correlating the average scores with the average scores for neuroticism, self‐evaluation, solitude, and mental well‐being. Based on Cohen (1992), correlation values around 0.10 were considered weak, around 0.30 were considered moderate, and around 0.50 were classified as strong.

Measurement invariance was examined across gender, different branches of learning and specialties, and birthplace using the total sample. Configural (weak), metric (medium), and scalar (strong) measurement invariance were assessed. Configural invariance implies the same number of factors and that the patterns of indicators loading on the factors are the same across groups (i.e., the unconstrained latent model should fit the data well in both groups). Metric invariance places equality restrictions on the magnitude of the loadings across groups, while scalar invariance imposes equality restrictions on both the item loadings and item intercepts across groups. According to the recommendations of Cheung and Rensvold (2002) and Chen (2007), the change (Δ) of fit indices between two adjacent invariance levels—that is, ΔCFI ≤ 0.010 and ΔRMSEA ≤ 0.015 or ΔSRMR ≤ 0.010—was used as evidence of metric or scalar invariance.

The number of latent classes was determined by comparing the model fit indices, including Akaike information criterion (AIC), Bayesian information criterion (BIC), and sample size adjusted BIC (SSA‐BIC), with smaller values representing better fit (Masyn 2013). Additionally, the Lo–Mendell Rubin adjusted likelihood ratio test (LMR‐LRT) and the bootstrapped likelihood ratio test (BLRT) were employed to evaluate whether a model exhibits significant improvement from the model with *k* − 1 classes to *k* classes, with significance indicating *k* classes having a better good fit (John et al. 2014; Marsh et al. 2009). Greater entropy value indicates a higher probability of being able to classify participants into the latent class successfully (Masyn 2013; Tein et al., 2013). Values above 0.80 indicate classification with minimal uncertainty. Model identification involved evaluating 1000 sets of random starting values for all models and retaining 100 iterations for the final stage of optimization.

## 4 Results

### 4.1 Item Analysis

#### 4.1.1 EFA of 18‐Item Version

EFA was conducted on the first half of the sample from Cohort 1 (*n* = 1102). The Kaiser–Meyer–Olkin (KMO) measure of sampling adequacy was determined using SPSS27.0 to be 0.952, which indicates a sufficient sample size for conducting factor analysis (Klein and Alan Dabney 2013). Bartlett's test of sphericity (in SPSS27.0) yielded statistically significant results (13578.704, *p <* 0.001), providing additional support for the adequacy of the sample (Klein and Alan Dabney 2013). The scores obtained from the 18‐item CPP were subjected to EFA with parallel analysis to identify underlying factors using Mplus 8.0. The results of the parallel analysis provided support for a three‐factor structure (empirical eigenvalues: F1 = 9.348, F2 = 1.889, F3 = 1.055, and F4 = 0.705; simulated eigenvalues: F1 = 1.233, F2 = 1.192, F3 = 1.157, and F4 = 1.127). However, the factor loading of one item was below 0.4, and this item was accordingly discarded (“In order not to feel anxious or guilty, I will take the initiative to apologize after quarreling with others, even if it is not my fault”). The other four items were also deleted for cross loading issues, as the absolute difference in factor loadings between the two items is less than 0.40, see Table 1 (Matsunaga 2010). These four items were “If I stopped putting others' needs ahead of my own, people would no longer like me,” “It is very difficult for me to express criticism even if it is constructive because I don't want to make anyone angry with me,” “I must never let other people down by failing to do everything they expect of me even when I know that the demands are excessive or unreasonable,” and “To avoid standing out or appearing non‐conformist, I tend to remain silent when I find myself in disagreement with others, whether it be in terms of thoughts or actions.” Finally, three factors were extracted from 13 items, which explained 67% of the variance.

**TABLE 1:** Factor loadings of the 18‐item CPP.

	Factor 1	Factor 2	Factor 3
Item 1	0.637	0.059	0.07
Item 2	0.898	−0.016	−0.058
Item 3	0.867	0.033	0.005
Item 4	0.365	0.506	0.007
Item 5	0.097	0.679	0.080
Item 6	0.069	0.875	−0.073
Item 7	0.101	0.784	0.007
Item 8	−0.1	0.937	−0.013
Item 9	−0.07	0.676	0.278
Item 10	0.005	0.686	0.194
Item 11	−0.042	0.173	0.525
Item 12	0.041	0.05	0.682
Item 13	0.02	−0.001	0.830
Item 14	0.013	−0.036	0.871
Item 15	0.000	0.163	0.716
Item 16	−0.066	−0.024	0.605
Item 17	0.003	0.218	0.569
Item 18	0.063	0.310	0.378

#### 4.1.2 CFA of 13‐Item Version

CFA was conducted on the second half of the sample (*n* = 1101). The majority of the goodness of fit indices for the three‐factor structure of 13‐item CPP indicated satisfactory fit (*χ* ^2^ = 357.178, df = 62, *χ* ^2^/df = 5.761, *p <* 0.001, CFI = 0.966, TLI = 0.957, RMSEA = 0.066 with 90% CI range from 0.060 to 0.073, SRMR = 0.040). As shown in Table 2, all items exhibited standardized factor loading ranging from 0.492 to 0.860, which exceeded the cutoff value of 0.40 (Stevens 2001). There were statistically significant correlations between the three latent variables (*p <* 0.001). The level of TPP thought exhibited a strong positive correlation with both TPP behavior (*r* = 0.786) and TPP feeling (*r* = 0.537). Additionally, there was a significant positive correlation between TPP behavior and TPP feeling (*r* = 0.613).

**TABLE 2:** Standardized factor loading of the 13‐item CPP.

	Thought	Behavior	Feeling
Item 1	0.721		
Item 2	0.843		
Item 3	0.854		
Item 4		0.739	
Item 5		0.848	
Item 6		0.860	
Item 7		0.859	
Item 8		0.806	
Item 9			0.677
Item 10			0.810
Item 11			0.849
Item 12			0.780
Item 13			0.492

Abbreviation: CPP, Chinese people‐pleasing questionnaire.

As the correlation between TPP thought and TPP behavior was high, a two‐factor structural model was further explored by combining TPP thought and TPP behavior as one dimension, with TPP feeling as the other dimension. This exhibited a significant positive correlation (*r* = 0.630) between the merged dimension and TPP feeling; however, the overall fit of the model was inadequate (*χ* ^2^ = 773.731, df = 64, *χ* ^2^/df = 12.090, *p <* 0.001, CFI = 0.918, TLI = 0.900, RMSEA = 0.101 with 90% CI [0.095 ~ 0.108], SRMR = 0.049). This finding provides additional support for the three‐factor structure.

For Cohort 2, the three‐factor structure of the 13‐item CPP also demonstrated a good fit (*χ* ^2^ = 600.489, df = 62, *χ* ^2^/df = 9.69, *p <* 0.001, CFI = 0.970, TLI = 0.962, RMSEA = 0.064, 90% CI = 0.060–0.069, SRMR = 0.035).

### 4.2 Reliability

The internal consistency of the 13‐item CPP was assessed using Cronbach's alpha, which demonstrated satisfactory reliability. In Cohort 1, the Cronbach's alpha coefficients were 0.854, 0.917, and 0.857 for the thought, behavior, and feeling dimensions, respectively, for the whole sample. For Cohort 2, the Cronbach's alpha coefficients were 0.838, 0.922, and 0.858, for thought, behavior, and feeling, which demonstrated strong internal consistency. McDonald's *ω* values exceeded 0.85 for the three subscales (thought *ω* = 0.859, behavior *ω* = 0.918, and feeling *ω* = 0.864), demonstrating high reliability. In Cohort 2, the McDonald's *ω* coefficients for the three subscales also exceeded 0.85 (0.847, 0.924, and 0.863 for thought, behavior, and feeling, respectively).

The test–retest reliability scores were 0.570, 0.629, and 0.614 for thought, behavior, and feeling, respectively. The intra‐class correlation coefficients were found to be 0.726, 0.772, and 0.761 for thought, behavior, and feeling, respectively (0.750–0.900 representing good reliability) (Koo and Li 2016). Overall reliability was considered satisfactory except for the moderate reliability observed in TPP thought.

### 4.3 Measurement Invariance

As shown in Table 3, the results from all indices indicated that configural, metric, and scalar invariances were supported across gender (male vs. female), different branches of learning and specialties (arts or science), different birthplaces (city vs. rural areas) and different time points (Cohort 1 and Cohort 2).

**TABLE 3:** Measurement invariance of the CPP across groups in the total sample.

Group (N)		CFI	TLI	RMSEA (90% CI)	SRMR	ΔCFI	ΔTLI	ΔRMSEA	ΔSRMR
Males (1181) Female (964)	Male	0.968	0.960	0.066 (0.060–0.073)	0.035				
	Female	0.967	0.958	0.066 (0.059–0.073)	0.042				
	Configural	0.967	0.958	0.067 (0.062–0.072)	0.039	—	—	—	—
	Metric	0.967	0.962	0.064 (0.060–0.069)	0.047	0.000	0.004	0.001	0.003
	Scalar	0.964	0.962	0.064 (0.060–0.068)	0.048	0.003	0.000	0.000	0.001
Arts (1016) Science (1129)	Science	0.969	0.961	0.065 (0.059–0.072)	0.037				
	Art	0.967	0.958	0.067 (0.060–0.074)	0.042				
	Configural	0.968	0.959	0.067 (0.062–0.071)	0.039	—	—	—	—
	Metric	0.968	0.963	0.063 (0.059–0.068)	0.045	0.000	0.004	0.004	0.006
	Scalar	0.967	0.965	0.061 (0.057–0.065)	0.043	0.001	0.002	0.002	0.002
City (1140) Rural (1041)	City	0.970	0.962	0.065 (0.059–0.072)	0.037				
	Rural	0.964	0.954	0.069 (0.062–0.076)	0.041				
	Configural	0.967	0.957	0.068 (0.063–0.073)	0.039	—	—	—	—
	Metric	0.966	0.961	0.065 (0.060–0.069)	0.045	0.001	0.004	0.003	0.006
	Scalar	0.966	0.964	0.062 (0.058–0.067)	0.046	0	0.003	0.003	0.001
Different time points	Time point 1	0.970	0.962	0.063 (0.059–0.068)	0.037				
	Time point 2	0.970	0.962	0.064 (0.060–0.069)	0.035				
	Configural	0.970	0.962	0.064 (0.061–0.068)	0.036				
	Metric	0.970	0.965	0.061 (0.058–0.065)	0.038	0.000	0.003	0.003	0.002
	Scalar	0.969	0.968	0.059 (0.056–0.062)	0.039	0.001	0.003	0.002	0.001

Abbreviation: CPP, Chinese people‐pleasing questionnaire.

### 4.4 Convergent and Criterion‐Related Validity

To evaluate the validity of the scores, we conducted a comprehensive analysis of bivariate correlations between the 13‐item CPP scores across three subscales and the total TPP score, which ranged from 0.873 to 0.903. Additionally, this study calculated the AVE and SQRAVE. The AVE was 0.65 (exceeding the 0.5 threshold), and the SQRAVE was 0.81, both of which were higher than the correlations among the three latent variables, indicating strong convergent and discriminant validity (Hair et al. 2017). Furthermore, we examined the correlations between the 13‐item CPP subscale scores and other measures. The results revealed significant positive associations between higher TPP and increased levels of neuroticism, social avoidance, and loneliness (see Table 4), while showing negative relationships with self‐evaluation. TPP behavior and feeling were also negatively related to openness, conscientiousness, and extroversion.

**TABLE 4:** Correlation among CPP scores and all other measures.

	Whole sample (*n* = 2203)			
	TPP thought	TPP behavior	TPP feeling	TPP total
TPP thought	1			0.793**
TPP behavior	0.674**	1		0.903**
TPP feeling	0.461**	0.580**	1	0.830**
Openness	−0.022	−0.156**	−0.240**	−0.182**
Agreeableness	0.024	−0.152**	−0.027	−0.075**
Conscientiousness	−0.004	−0.219**	−0.123**	−0.156**
Extroversion	−0.016	−0.129**	−0.203**	−0.152**
Neuroticism	0.247**	0.361**	0.433**	0.424**
Social avoidance	0.127**	0.220**	0.351**	0.289**
Loneliness	0.284**	0.370**	0.360**	0.406**
Self‐evaluation	−0.185**	−0.386**	−0.339**	−0.377**
Mental well‐being	−0.086**	−0.252**	−0.249**	−0.248**

Abbreviations: CPP, Chinese people‐pleasing questionnaire; TPP, tendency for people‐pleasing.

** *p <* 0.001.

### 4.5 Predictive Validity of the CPP

The predictive validity of the 13‐item CPP was examined by assessing its relationship with students' mental well‐being, using a validated mental well‐being scale. The results revealed a significant negative correlation between higher TPP thought behaviors and feelings and lower mental well‐being, consistent with previous findings by Ehman (2021). Specifically, individuals who exhibited more pronounced TPP‐related thought, patterns and emotional responses tended to report lower levels of mental well‐being. This suggests that individuals with higher TPP may experience greater psychological distress or difficulty in achieving positive mental health outcomes. These findings align with the theoretical understanding that excessive people‐pleasing behaviors, as measured by the 13‐item CPP, are often linked to adverse emotional and psychological consequences (Ehman 2021). Overall, the negative relationship between TPP and mental well‐being supports the construct's validity as a predictor of psychological health outcomes.

### 4.6 Determination of the Latent Classes

LPA was conducted on the means of the three 13‐item CPP sub‐dimensions. The LPA fit indices, ranging from two to six classes, are presented in Table 5. An improvement in the AIC, BIC, SSA‐BIC (decrease in terms of values), and entropy was observed up to the seven‐class solution. The *p* values for the BLRTs were significant for all six solutions. However, for the LMR‐LRT results, the *p* was above 0.05 for the five‐class solution, which indicates that the improvement in model fit from the four‐class to the five‐class solution was not significant at the 0.05 level; the four‐class solution was therefore chosen. The entropy (0.798) and the most likely profile were found to be 0.830, 0.850, 0.860, and 0.932 for the four classes—meeting the cutoff recommended by Weller et al. (2020).

**TABLE 5:** Model fit information for latent profile analysis of timepoint 1 (*N* = 2145).

	AIC	BIC	SSA‐BIC	BLRT	LMR‐LRT	Entropy	Sample size	Proportions	Classification probabilities for the most likely latent class membership					
									1	2	3	4	5	6
2 class	17348.521	17405.230	17373.459	1482.856 *p* < 0.001	1436.054 *p* < 0.001	0.732	Class1 = 887	41.352%	0.903	0.097				
							Class 2 = 1258	58.648%	0.062	0.938				
3 class	16860.014	16939.407	16894.927	496.507 *p* < 0.001	480.836 *p* < 0.001	0.726	Class 1 = 498	23.217%	0.876	0.124	0.000			
							Class 2 = 1125	52.448%	0.050	0.869	0.081			
							Class 3 = 522	24.336%	0.000	0.124	0.876			
4 class	16510.217	16612.293	16555.104	346.505 *p* < 0.001	357.798 *p* < 0.001	0.798	Class 1 = 798	37.203%	0.850	0.038	0.000	0.112		
							Class 2 = 301	14.033%	0.170	0.830	0.000	0.000		
							Class 3 = 74	3.450%	0.000	0.000	0.860	0.140		
							Class 4 = 972	45.315%	0.062	0.000	0.007	0.932		
5 class	16264.568	16389.327	16319.431	253.649 *p* < 0.001	245.643 *p* = 0.120 > 0.05	0.879	Class 1 = 414	19.301%	0.943	0.000	0.057	0.000	0.000	
							Class 2 = 402	18.741%	0.000	0.914	0.000	0.006	0.080	
							Class 3 = 593	27.646%	0.045	0.000	0.913	0.000	0.042	
							Class 4 = 59	2.751%	0.000	0.059	0.000	0.941	0.000	
							**Class 5 = 677**	**31.562%**	**0.000**	**0.034**	**0.050**	**0.000**	**0.916**	
6 class	16061.097	16208.541	16125.936	211.47 *p* < 0.001	204.796 *p* = 0.100 > 0.05	0.883	Class 1 = 356	16.597%	0.938	0.022	0.040	0.000	0.000	0.000
							Class 2 = 83	3.869%	0.130	0.712	0.158	0.000	0.000	0.000
							Class 3 = 575	26.807%	0.020	0.015	0.920	0.000	0.044	0.000
							Class 4 = 402	18.741%	0.000	0.000	0.000	0.923	0.071	0.006
							**Class 5 = 669**	**31.189%**	**0.000**	**0.000**	**0.044**	**0.034**	**0.922**	**0.000**
							Class 6 = 60	2.797%	0.000	0.000	0.000	0.053	0.000	0.947

*Note:* Information for the best fitting model is in bold.

The differences of the three 13‐item CPP sub‐dimensions at the two time points were compared using one‐way repeated measures analysis of variance (RANOVA) for the four classes. According to Mauchly's Test of Sphericity (*p >* 0.05), the variance between different groups is homogeneous. All within‐subject and between‐subject *F* values yielded statistically significant results, providing further evidence for profile identification for different time points (refer to Table 6).

**TABLE 6:** One‐way RANOVA results of four profiles on sub‐dimensions of TPP (Mean ± SD).

	Thought		Behavior		Feeling	
	Time point 1	Time point 2	Time point 1	Time point 2	Time point 1	Time point 2
Profile 1	1.49 ± (0.57)	1.96 ± (0.97)	1.12 ± (0.23)	1.52 ± (0.85)	2.16 ± (0.92)	2.45 ± (0.96)
Profile 2	2.93 ± (0.79)	2.84 ± (0.9)	1.98 ± (0.48)	2.15 ± (0.86)	3.17 ± (0.85)	3.15 ± (0.92)
Profile 3	3.72 ± (0.63)	3.47 ± (0.78)	3.38 ± (0.49)	3.10 ± (0.84)	3.80 ± (0.64)	3.66 ± (0.78)
Profile 4	5.14 ± (0.68)	4.38 ± (1.18)	4.96 ± (0.56)	3.97 ± (1.24)	4.99 ± (0.85)	4.36 ± (1.15)

Within‐subject	*F*	*F*	*F*
2 time points	21.75**	31.76**	17.50**
Time points*4 groups	54.42**	92.17**	30.07**
Between‐subject	859.46***	1512.65***	405.66**
Partial Eta square	0.56	0.69	0.37

*Note:* **p < 0.01; ***p < 0.001.

Abbreviation: TTP, tendency for people‐pleasing.

**FIGURE 1:**
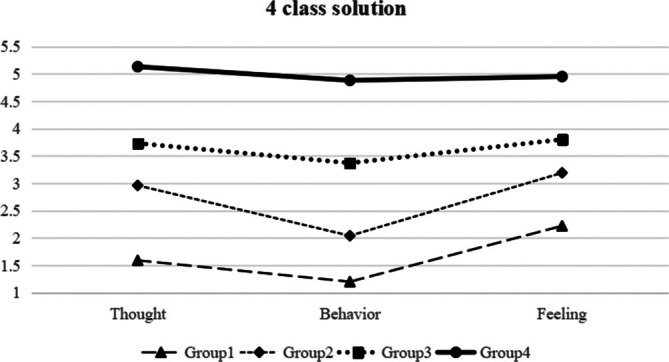
Parameter estimates for the four‐profile model.

As seen in Figure 1, the first class, referred to as “No TPP,” accounted for 14% (*n* = 301) of the students and exhibited significantly low levels in each dimension (mean scores < 2). The second class, referred to as “Slight TPP,” encompassed 37% (*n* = 798) of the students and exhibited markedly diminished levels across all mean scores (hovering around 3). The third profile, referred to as “Moderate TPP,” encompassed 45.3% (*n* = 972) of the students and exhibited significantly low levels across all dimensions (3 < mean scores < 4). The fourth profile, referred to as “Serious TPP,” encompassed 3.45% (*n* = 74) of the student population and was distinguished by exceptionally elevated levels across all dimensions (mean scores > 4.9). The results were similar to those from YouGov's investigation, who found half (49%) of Americans would self‐identify as people‐pleasers, including the 14% who said they “definitely” would.

Table 7 presents the cross tabulation of the three demographic variables and four types of TPP. The chi‐square test was statistically significant only for gender; for males, 48.8% identified as Moderate TPP, which was greater than among female respondents (41.1%) More female respondents (35.4%) identified as Slight TPP than males (35.4%). These results were inconsistent with the YouGov survey, which found women were more likely to be a people‐pleaser. One reason might be that the present sample consisted of first‐year university students, while YouGov used adults; another possible reason would be that our classification was built on our cutoff value and severity level, while in the YouGov survey, they asked people to self‐identify as being a people‐pleaser based on only one self‐reported item.

**TABLE 7:** Cross tabulation of demographic variable × TPP types.

Group (*N*)	No TPP	Slight TPP	Moderate TPP	Serious TPP	Pearson chi‐square
Male (1181)	140 (12%)	418 (35%)	576 (49%)	47 (4%)	19.92**
Female (963)	160 (17%)	380 (39%)	396 (41%)	27 (3%)	
Science (1129)	161 (14%)	402 (36%)	531 (47%)	35 (3%)	4.16
Arts (1016)	139 (14%)	396 (39%)	441 (43%)	39 (4%)	
Urban (1140)	160 (14%)	407 (36%)	493 (43%)	44 (4%)	2.60
Rural (1041)	140 (13%)	391 (38%)	479 (46%)	30 (3%)	

Abbreviation: TTP, tendency for people‐pleasing.

** *p* < 0.01.

## 5 Discussion

In this study, we adapted Jiang's translated 24‐item CPP (yes or no) into a 13‐item version and evaluated its psychometric properties for the first time. The test retained three dimensions: thought (3 items), behavior (5 items), and feelings (5 items). The CFA of the 13‐item CPP confirmed a three‐factor structure, which is consistent with Braiker's original study design. Moreover, each latent factor demonstrated significant loadings on the overall 13‐item CPP factor. Empirical evidence is thus provided supporting the reliability, internal structure, as well as the convergent and discriminant validity of the 13‐item CPP. In addition to CFA, multi‐group CFA was employed to examine measurement invariance. The 13‐item CPP also demonstrated strong measurement invariance across gender, academic tracks, and birthplace at the configural, metric, and scalar levels. This implies that the multidimensional structure of the scale is equally applicable in Chinese male and female samples, arts and science samples, and urban and rural areas. It should be noted that there was longitudinal measurement invariance between the two time points. The longitudinal invariance of the scores indicates whether future researchers implementing tracking studies of college students' people‐pleasing can use the 13‐item CPP to evaluate their level of people‐pleasing at different time points. These cross‐group and cross‐time stability highlight the universality of people‐pleasing behaviors, despite cultural variations in their expression and social reinforcement. Future research should explore how sociocultural factors influence the development and reinforcement of people‐pleasing tendencies, particularly in educational and workplace settings.

The item analysis revealed a high level of internal consistency and reliability for the scale, with satisfactory Cronbach's alphas (> 0.8) for both the overall scale and its three sub‐scales. It is worth noting that Cronbach's alpha can be influenced by the number of items (Streiner 2003). Additionally, the composite reliability, assessed through McDonald's omega, demonstrated good reliability (all *ω*s > 0.85). The test–retest reliability was assessed using ICC due to correlation issues. The ICC values for the entire scale and two sub‐scales demonstrated good reliability (ICC > 0.75). However, the thought subscale showed slightly lower reliability, likely due to its limited number of items, as it only consisted of three items. Construct validity was established through a series of correlations, which demonstrated that CPP scores exhibited positive associations with neuroticism, social avoidance, and loneliness. These findings align with those of previous research (Huntington 2023). Additionally, the results indicated a negative relationship between CPP scores and self‐evaluation, which supports the notion that individuals with high levels of people‐pleasing lack an internal compass to assess the value of their own actions (Ehman 2021; Svoboda 2008). The negative correlation between CPP scores and participants' mental health, assessed 1 month later, further corroborates existing literature (Feely et al. 2007; Huntington 2023). These findings address questions 1 and 2, thus demonstrating that the modified CPP is suitable for use in the Chinese context and displays robust psychometric properties. Additionally, the strong associations between people‐pleasing tendencies and adverse mental health outcomes highlight the psychological burden of excessive people‐pleasing. The negative correlations with self‐evaluation and mental well‐being suggest that individuals with heightened people‐pleasing tendencies may experience challenges in emotional regulation, increasing their susceptibility to anxiety and depression. This underscores the importance of early psychological interventions to help individuals develop healthier interpersonal boundaries and prevent long‐term mental health issues.

LPA identified four distinct profiles of students at two different time points, varying in the people‐pleasing severity level. These profiles have been named from no TPP to serious TPP based on their characteristics, with specific cutoffs that can be utilized in future studies to identify students who may be experiencing serious TPP and potentially related to mental health issues. The LPA findings further underscore the complexity of people‐pleasing behaviors. The identification of distinct subgroups suggests that people‐pleasing exists on a spectrum, with some individuals exhibiting mild tendencies, while others experience more severe patterns associated with significant psychological distress. The existence of a “serious TPP” group, though small, indicates that a subset of individuals may require targeted clinical interventions. These findings highlight the need for a nuanced approach to addressing people‐pleasing tendencies, incorporating both preventative strategies for those with mild tendencies and therapeutic interventions for those in distress. Future studies should examine the long‐term stability of these latent profiles and explore potential factors that influence transitions between them, such as life stressors or changes in social environments. It is also important to note that while the 13‐item CPP is not intended as a diagnostic tool, it can provide valuable insights for cohorts studying university student mental health. These results address question 3 and indicate that LPA can be employed to establish cutoff criteria for identifying individuals exhibiting TPP.

This study provides valuable insights into the mental health risks associated with people‐pleasing behaviors among Chinese university students. By validating the Chinese People‐Pleasing questionnaire and identifying distinct latent profiles, the research highlights the urgent need for mental health interventions tailored to students with moderate to severe people‐pleasing tendencies. The significant negative correlations between CPP scores and mental well‐being underscore the psychological cost of excessive people‐pleasing, which manifests as neuroticism, social withdrawal, and diminished self‐worth. As people‐pleasing behaviors are deeply embedded in the collectivist cultural framework, mental health interventions must incorporate culturally relevant approaches to address this pervasive issue. The 13‐item CPP offers a practical tool for identifying at‐risk students and promoting proactive mental health strategies that empower individuals to prioritize their well‐being without compromising their interpersonal relationships.

## 6 Implications

The findings of this study have both theoretical and practical implications. Theoretically, the validation of the Chinese People‐Pleasing questionnaire (CPP) advances the cross‐cultural understanding of people‐pleasing behaviors and their psychological impact. It provides empirical support for the three‐factor model and highlights the role of people‐pleasing tendencies in mental health outcomes. Practically, the 13‐item CPP questionnaire can serve as a valuable screening tool in educational and clinical settings, enabling mental health practitioners to identify individuals at risk of negative psychological outcomes due to excessive people‐pleasing behaviors. Additionally, the identification of distinct latent profiles offers insights into personalized intervention strategies aimed at fostering healthier interpersonal boundaries and self‐concept among students. These advancements align with contemporary efforts to integrate culturally sensitive tools into mental health frameworks while addressing the nuanced needs of at‐risk populations.

## 7 Limitations and Future Research Directions

The current study has significant strengths that deserve highlighting, including employing McDonald's *ω* to assess internal consistency and examining longitudinal measurement invariance. Additionally, we have made a valuable contribution to existing research on the cross‐cultural validity of the 13‐item three‐structure CPP for assessing TPP in Chinese context. However, it is imperative to acknowledge several limitations in our study. The inclusion of only university students restricts the generalizability of our findings. Further research is required to validate the applicability of the 13‐item CPP in diverse populations. The 13‐item CPP is a concise questionnaire specifically designed for research purposes in university samples to serve as an indicator of TPP rather than providing diagnostic assessments for personality disorders. Further investigation is required to ascertain the applicability of the CPP within clinical settings. Third, culture may play a role in students' TPP. Being part of a collectivist Confucian culture, which places more emphasis on harmonious coexistence, students often cultivate geniality and empathy, which might be one cause of TPP that would be worth exploring in the future. The expression and distribution of behaviors, as well as the validation of the 13‐item CPP, have only been confirmed within a representative sample of college students from one province in China. Therefore, future cross‐cultural comparisons and validations are necessary to assess the generalizability of the CPP across different contexts.

## 8 Conclusion

By modifying Jiang's 24‐item CPP into a 13‐item version, this study successfully confirmed its three‐factor model (thought, behavior, and feeling). The overall CPP score exhibited strong internal consistency and good construct validity. Measurement invariance analysis revealed consistent results across gender, academic disciplines, birthplace, as well as strong longitudinal invariance. These findings support the use of the 13‐item CPP as an assessment tool within the Chinese cultural context. A threshold was also determined using LPA to identify students displaying TPP, which indicates the potential clinical utility of the 13‐item CPP. The findings also indicate that higher levels of TPP thought patterns and emotional responses are associated with lower mental well‐being, highlighting the negative impact of people‐pleasing tendencies on psychological health and reinforcing the importance of addressing these behaviors in mental health interventions.

## Ethics Statement

The study was conducted in accordance with the Declaration of Helsinki and approved by the Ethics Committee of Dongguan University of Technology (Protocol code: HR011‐2022; Date of approval: 20 April 2022).

## Consent

Informed consent was obtained from all subjects involved in the study.

## Conflicts of Interest

The authors declare no conflicts of interest.
